# High dose rate interstitial brachytherapy for early stage lip cancer using customized dental spacer

**DOI:** 10.1093/jrr/rraa019

**Published:** 2020-04-08

**Authors:** Koji Masui, Hideya Yamazaki, Gen Suzuki, Daisuke Shimizu, Kanako Kawabata, Naoki Noguchi, Tadashi Takenaka, Ken Yoshida, Naoya Murakami, Masayuki Naito, Toshiro Yamamoto, Narisato Kanamura, Satoshi Komori, Akifumi Oshita, Jun Asai, Kei Yamada

**Affiliations:** 1 Department of Radiology, Kyoto Prefectural University Graduate School of Medical Science, Kyoto, Japan; 2 Department of Radiation Oncology, Osaka Medical College, Takatsuki, Osaka, Japan; 3 Department of Radiation Oncology, National Cancer Center Hospital, Tokyo, Japan; 4 Department of Dental Medicine, Kyoto Prefectural University Graduate School of Medical Science, Kyoto, Japan; 5 Department of Dermatology, Kyoto Prefectural University Graduate School of Medical Science, Kyoto, Japan

**Keywords:** lip cancer, HDR, interstitial, brachytherapy, dental customized spacer

## Abstract

The present study aimed to report the efficacy and toxicity of our high-dose-rate (HDR) brachytherapy for early stage lip cancer (LC) using customized dental spacers. A retrospective analysis was performed among six patients with early stage LC treated with HDR interstitial brachytherapy between April 2015 and August 2019 using customized dental spacers. The total treatment dose was 49 Gy/7 fractions or 54 Gy/9 fractions. The median follow-up duration for the patients was 13 (range: 2–52) months. All patients completed the entire brachytherapy protocol safely and have experienced no local recurrence thus far. The CTV D100 and D90 values per fraction were median 100 (range: 98.3–100) % prescribed dose (PD) and median 133.4 (range: 129.3–138.9) % PD, respectively. The D2cc and D0.1cc values per fraction for the mandible were median 1.07 (range, 0.79–1.88) Gy and median 1.65 (range: 1.21–2.83) Gy, D2cc and D0.1cc values per fraction for oral cavity were median 1.48 (range, 1.31–1.72) Gy and median 2.73 (range: 1.79–2.88) Gy, respectively. Acute toxicities encountered were mucositis and lip edema limited to the irradiated area; none of them was beyond grade 2 and all were resolved within 1–2 months after treatment. We did not observe any late grade 2 adverse events or worse. This study shows that the adverse effects of HDR brachytherapy for early stage LC can be minimized using a dental spacer. Cooperation with the dentistry department is essential to make spacers that are individually customized for each patient.

## INTRODUCTION

Lip cancer (LC) is a rare tumor, particularly in Eastern Asia, with a reported incidence rate of only 1.5% among all cases of oral and pharyngeal cancer [1]. The same is true for Japan. LC is common in the elderly population and mainly affects the lower lip.

Available treatment modalities include surgery and radiotherapy, when LC is found in its early stages (T1/T2N0). Surgery yields excellent outcomes but with less satisfactory functional and cosmetic outcomes and is not indicated for elderly patients who are unable to tolerate general anesthesia. Radiotherapy is known to yield excellent outcomes not only with local tumor control but also with both functional and cosmetic results [2–7]. Although radiotherapy can cause complications, such as oral mucositis, xerostomia and osteoradionecrosis, brachytherapy could reduce their incidence by delivering high-dose radiation to the tumor while sparing the surrounding normal tissue. In fact, low-dose-rate (LDR) and high-dose-rate (HDR) brachytherapy have been performed for patients with LC [2–7]. Compared with LDR brachytherapy, HDR brachytherapy does not require an isolation ward and can be performed as a minimally invasive treatment using local anesthesia and minimal sedation [8]. We therefore treated patients with LC using HDR brachytherapy, with a customized dental spacer created in collaboration with the department of dentistry. This study aimed to assess the efficacy and toxicity of our HDR brachytherapy for early stage LC using a customized dental spacer.

## MATERIALS AND METHODS

### Patients

This single-institution retrospective analysis included six patients diagnosed with LC (squamous cell carcinoma) at early stages (T2N0M0) (Table 1). All the patients were treated with HDR brachytherapy alone between April 2015 and August 2019. LC was staged by physical examination of the extra- and intra-oral regions, using Lugol’s staining method to visualize the infiltrated area, in conjunction with ultrasonography (prosound α7; Hitachi Aloka Medical, Tokyo, Japan) and positron emission tomography–computed tomography (CT). Before planning of brachytherapy, each patient was referred to the department of dentistry at our hospital to undergo a general dental check-up and to make a customized dental spacer.

**Table 1:** Patients’ characteristics

Factors		
Median age (years)		79 (60–92)
Sex	Male	4
	Female	2
T	Tl	0
	T2	6
N	NO	6
	Nl	6
Tumor volume (ml)		4.4 (range: 1.2–8.2)
Location of tumor	Lower lip	6
	Upper lip	0
Dose (Gy)	49 Gy/7 fractions	4
	54 Gy/9 fractions	2
Implant needles		2–6

All patients provided informed consent for inclusion in the study. The study protocol was approved by the ethical committee of Kyoto Prefectural University of Medicine (approval number, ERB-C-1403).

### Fabrication of customized dental spacers

**Fig. 1.**
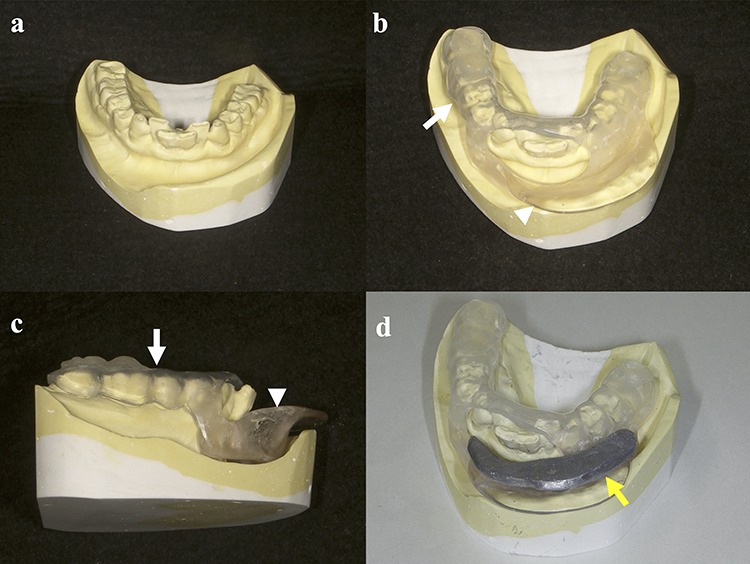
Schemata for the fabrication of spacers: (**a**) dental impression; (**b**, **c**) spacer made from a two-layer thermoforming plate (white arrow) and self-curing resin (white arrowhead); (**d**) spacer with shield made from lead (yellow arrow).

During the first dental visit, a radiation oncologist, dentist and dental technician examined the patients together, and discussed the size and shape of dental spacers, taking into account the tumor size and location. Afterward, the dentist took a dental impression as is done for normal dental treatment. Based on the discussion with the radiation oncologist and dentist, the dental technician made customized dental spacers. These spacers were made using a two-layer thermoforming plate made of copolyester (Erkoloc-pro; Erkodent Erich Kopp GmbH, Germany), polyurethane and self-curing resin. In addition to keeping radiation away from certain parts of the oral cavity (i.e. the tongue, gums, teeth and mandible), the spacers were adjusted once or twice to ensure that they could be easily attached and detached and that patients did not feel uncomfortable putting them on. Finally, removable shields made of lead were made and adjusted to fit the spacers. These lead shields were used only during the actual irradiation but not while the catheters were being placed. The entire process was completed in ~1–2 weeks (Fig. 1).

## Brachytherapy

### Interstitial brachytherapy

**Fig. 2.**
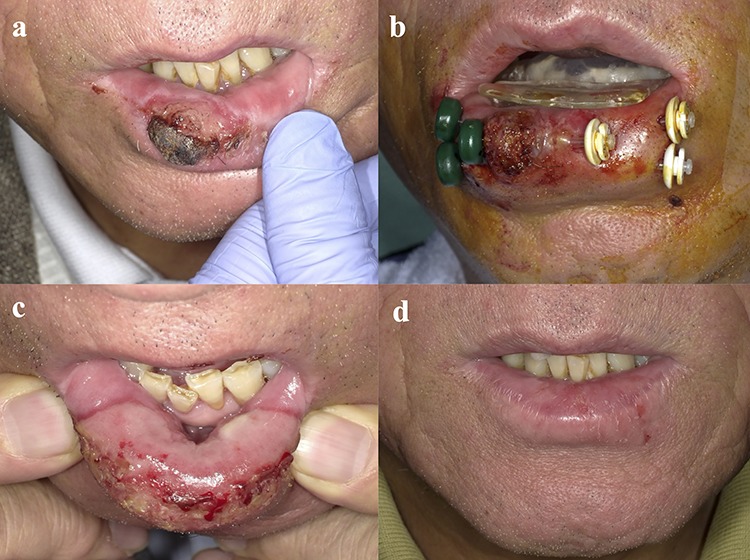
Photograph of patient with lower LC T2 SCC: (**a**) LC region before brachytherapy; (**b**) insertion of applicators; (**c**) 14 days after brachytherapy, acute mucositis on the lower lip observed only on the irradiated area; (**d**) 8 months after brachytherapy, the tumor resolved and no late complications occurred.

First, we attached the customized spacer without the lead shield, and we started to insert the interstitial needles. We implanted plastic applicators (OncoSmart® catheter system; Elekta AB-Nucletron, Stockholm, Sweden) in the tumors with 10-15 mm between them using the freehand technique under local anesthesia and minimum sedation in the operating room (Fig. 2a and b). After the catheters were inserted, we performed CT scans with the spacer attached to patients. These CT images were obtained with a 2.5-mm slice thickness.

### Treatment planning

We set the clinical target volume (CTV) to include the gross tumor volume and areas unstained with iodine, and added a 10 mm margin only laterally. No planning target volume margin was added around the CTV. Doses were calculated using the treatment planning system (Oncentra® Brachy; Elekta AB-Nucletron, Stockholm, Sweden). The total treatment dose was 49 Gy/7 fractions or 54 Gy/9 fractions (6 or 7 Gy per fraction). Our dosimetric goal was to cover the CTV with the prescribed dose (PD). We optimized the dose that covered 100% of the CTV [D100(CTV)] as the PD as much as possible. MicroSelectron HDR® ^192^Ir v3 (Elekta AB, Stockholm, Sweden) was used to administer treatment.

### Follow-up

First, a follow-up visit was planned 1–2 weeks after treatment, and then patients were evaluated every 1 month in the first 6 months. After that, they were evaluated every 2 months for the first 5 years after treatment.

## RESULTS

### Patients

The median follow-up duration for the patients was 13 (range, 2–52) months. All patients safely received the entire course of brachytherapy. All patients were able to continue with oral intake during treatment. All tumors disappeared completely with no evidence of recurrence at the time of reporting. One patient experienced ipsilateral cervical lymph node metastasis 13 months after treatment, which was salvaged by neck dissection, without disease recurrence. The prescription dose per fraction was 6 Gy, with two patients receiving 9 treatment fractions and 7 Gy and four patients receiving 7 treatment fractions. The CTV was 4.4 (range, 1.2–8.2) ml.

### Toxicity

Adverse events were assessed using the Common Terminology Criteria for Adverse Events version 4. All the acute toxicities encountered were grade 2 events or below. All patients experienced mucositis and edema of the lip only at the irradiated area. All symptoms improved within 1–2 months after treatment. We did not observe any late grade 2 adverse events or worse. The patients were observed to have mildly dry lips and mild depigmentation. No ulceration or osteoradionecrosis was found in any of our patients (Fig. 2c and d).

### Dosimetric analysis

**Fig. 3.**
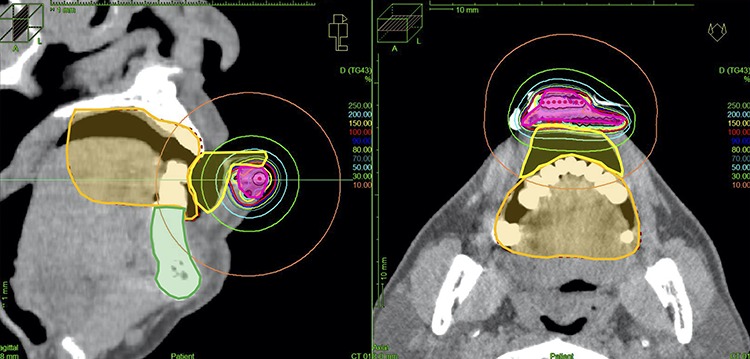
Treatment planning. The magenta line depicts the CTV, the orange line depicts the oral cavity, the green line depicts the mandible and the yellow line depicts a contour of the spacer. (Dose values were reference values because lead shield was used during actual treatment.)

The results of the dosimetric analysis are summarized in Table 2. The CTV D100, D98, D95 and D90 per fraction were median 100 (range: 98.3–100), 114.4 (range: 113.6–115.9), 122.9 (range: 120.9–126.1) and 133.4 (range: 129.3–138.9) % PD, respectively. The D2cc, D1cc and D0.1cc per fraction for the mandible were median 1.07 (range, 0.79–1.88), 1.24 (range: 0.91–2.18) and 1.65 (range: 1.21–2.83) Gy, respectively. The D2cc, D1cc and D0.1cc per fraction for the oral cavity were median 1.48 (range: .1.31–1.72), 1.90 (range: 1.47–2.17) and 2.73 (range: 1.79–2.88) Gy, respectively (these values were reference values because lead shield was used during actual treatment). A typical case is shown in Fig. 3.

**Table 2:** Dose parameters

Patient	Tumor volume (cc)	Dose/fractio (Gy)	Fractions	CTV				Mandible			Oral cavity		
				D100 (%PD)	D98	D95	D90	D2cc (Gy)	D1cc	D0.1cc	D2cc (Gy)	D1cc	DO.1 cc
1	8.2	6	9	100	114.8	123.5	133	1.33	1.49	1.8	1.72	2.17	2.86
2	6.5	7	7	98.3	113.7	122.3	133.7	1.86	2.18	2.83	1.5	1.89	2.6
3	6.1	6	9	100	114	121	129.5	1.31	1.52	1.96	1.54	2.06	2.88
4	1.2	7	7	99.7	115.9	124.7	135.6	0.83	0.98	1.5	1.45	1.9	2.87
5	1.9	7	7	100	113.6	120.9	129.3	0.81	0.92	1.21	1.31	1.47	1.79
6	2.8	7	7	100	115.7	126.1	138.9	0.79	0.91	1.22	1.42	1.7	2.4

## DISCUSSION

This is the first study to evaluate the efficacy and toxicity of HDR brachytherapy alone for LC with a customized dental spacer. There have been several attempts, including intraoral spacers for oral cancer [9, 10] and for LC [11, 12] to reduce adverse events resulting from radiotherapy. All cases of LC described in previous studies [9–12] were treated with external beam radiation and/or brachytherapy.

These previous studies obtained favorable results in preventing unnecessary radiation doses to adjacent normal tissue and reducing oral complications, and we achieved similar results (Figs 2c and 3). Our studies have shown that individually customized dental spacers may minimize the potential side-effects of radiation on oral tissues in LC patients.

There are several important points to note in using spacers: (i) keeping a sufficient distance between normal tissue and the irradiated site, (ii) attachments and detachments by radiation oncologists should be easy, and (iii) they should be comfortable for patients to wear during planning and treatment. In this study, because we cooperated with a dentist and dental technician in fabricating these spacers, we were able to make them to fit the abovementioned points. It is essential to make individual spacers that suit each patient, and we would like to thus strengthen the merit of cooperating with the dentistry department.

In HDR monotherapy for LC, several authors chose the dose schedule 4.5–6 Gy x 8–10 fractions [3–6]. However, none of them used image-guided brachytherapy. In this report, we treated all LC patients with image-guided brachytherapy and we needed to set a dose schedule and dosimetric goal. We applied the dose schedule (6 Gy x 9 fractions) and dosimetric goal (D100) that we usually used in image-guided brachytherapy for tongue cancer [13, 14], to brachytherapy for LC. Since we required two dose schedules (in 4 or 5 days) in order to complete the treatment in a week (excluding the weekend), we set 6 Gy x 9 fractions in 5 days or 7 Gy x 7 fractions in 4 days. The dose schedule was chosen according to the treatment schedule of each patient.

We optimized the dose that covered D100 as PD as much as possible. We could find no description of image-guided brachytherapy for LC in previous papers, but it is described as D95 = 100% of PD in a recent textbook [15]. Our results showed that D95 was 120–126% of PD. Although our target definition is smaller than the definition in this textbook, the dose we set might be too high. Currently, no side-effects that are problematic have been observed, nevertheless we need to be very careful about the course of side-effects in the future.

We also could not find the dose constraint of normal tissue in previous papers and recent textbooks. Our results showed that D2cc of the mandible and oral cavity was <2Gy and D0.1cc of them was <3Gy. However, as we used a lead shield in the actual treatment, these values were not accurate, and the actual dose to normal tissue was considered to be lower than those values. As our treatment planning system was not equipped with a system for inhomogeneity correction, we could not calculate exact values. Therefore, we described dose values to normal tissue as reference values.

We have experimentally measured how much doses were reduced with or without lead shield, using the spacers used to treat each patient. In that experiment we used an ^192^Ir source and radiophotoluminescent glass dosimeters (RPLDs). The results showed that the thickness of the lead shield was median 4.9 (range: 4.4–5.8) mm and the dose reduction rate was 70 (range: 66–76) %. This suggested that lead shield could significantly reduce the dose to normal tissues.

A limitation of this study was the retrospective observation and inclusion of only a small number of patients. A longer follow-up study with a larger sample size is needed before concrete conclusions can be reached, although the rare nature of LC may hinder the use of a prospective study or a large cohort study.

In conclusion, we were able to minimize the side-effects of HDR brachytherapy for early stage LC using individually customized dental spacers. Cooperation with the department of dentistry is essential to make spacers that fit each patient.

## CONFLICT OF INTEREST

None declared.
